# YBR/EiJ mice: a new model of glaucoma caused by genes on chromosomes 4 and 17

**DOI:** 10.1242/dmm.024307

**Published:** 2016-08-01

**Authors:** K. Saidas Nair, Mihai Cosma, Narayanan Raghupathy, Michael A. Sellarole, Nicholas G. Tolman, Wilhelmine de Vries, Richard S. Smith, Simon W. M. John

**Affiliations:** 1 Department of Ophthalmology, University of California, San Francisco, USA; 2 The Jackson Laboratory, Bar Harbor, ME, USA; 3 Howard Hughes Medical Institute, The Jackson Laboratory, Bar Harbor, ME, USA; 4 Department of Ophthalmology, Tufts University School of Medicine, Boston, MA, USA

**Keywords:** Mouse model, Glaucoma, Genetics, Ocular diseases

## Abstract

A variety of inherited animal models with different genetic causes and distinct genetic backgrounds are needed to help dissect the complex genetic etiology of glaucoma. The scarcity of such animal models has hampered progress in glaucoma research. Here, we introduce a new inherited glaucoma model: the inbred mouse strain YBR/EiJ (YBR). YBR mice develop a form of pigmentary glaucoma. They exhibit a progressive age-related pigment-dispersing iris disease characterized by iris stromal atrophy. Subsequently, these mice develop elevated intraocular pressure (IOP) and glaucoma. Genetic mapping studies utilizing YBR as a glaucoma-susceptible strain and C57BL/6J as a glaucoma-resistant strain were performed to identify genetic loci responsible for the iris disease and high IOP. A recessive locus linked to *Tyrp1^b^* on chromosome 4 contributes to iris stromal atrophy and high IOP. However, this is not the only important locus. A recessive locus on YBR chromosome 17 causes high IOP independent of the iris stromal atrophy. In specific eyes with high IOP caused by YBR chromosome 17, the drainage angle (through which ocular fluid leaves the eye) is largely open. The YBR alleles of genes on chromosomes 4 and 17 underlie the development of high IOP and glaucoma but do so through independent mechanisms. Together, these two loci act in an additive manner to increase the susceptibility of YBR mice to the development of high IOP. The chromosome 17 locus is important not only because it causes IOP elevation in mice with largely open drainage angles but also because it exacerbates IOP elevation and glaucoma induced by pigment dispersion. Therefore, YBR mice are a valuable resource for studying the genetic etiology of IOP elevation and glaucoma, as well as for testing new treatments.

## Abstract (precis)

**Summary:** We identify the YBR/EiJ mouse strain as a new model of high intraocular pressure and glaucoma, and also identify genetic loci that contribute to this glaucoma.

## INTRODUCTION

Glaucoma is a heterogeneous group of diseases characterized by death of retinal ganglion cells, specific visual field deficits and optic nerve degeneration. It is a leading cause of blindness worldwide and affects over 70 million people (Quigley, 1996). Elevated intraocular pressure (IOP) is often associated with glaucoma and is one of the strongest known risk factors (Gordon et al., 2002). Normal IOP is controlled by a balance between aqueous humor production by the ciliary body and its drainage through the trabecular and uveoscleral drainage pathways (Fautsch and Johnson, 2006; Shields, 1992). Decreased aqueous humor drainage is believed to be responsible for elevation of IOP in most cases. Experimentally elevating IOP can cause glaucoma in animals, supporting a harmful role for elevated IOP in human glaucoma (Morrison et al., 1990; Quigley et al., 1980).

Some autosomal dominant and recessive mutations causing glaucoma have been uncovered (Liu and Allingham, 2011). However, the mutations important for IOP elevation and susceptibility to neurodegeneration in most individuals with glaucoma are largely unknown. Glaucoma, in general, is thought to be a complex disease. The disease outcome in most glaucoma cases does not depend on a single genetic mutation but is instead controlled by mutations in multiple genes. The underlying complexity makes it difficult to identify glaucoma genes. Recent advances in genome-wide studies have led to the identification of multiple genes associated with glaucoma in humans (Bailey et al., 2016; Burdon et al., 2011; Ramdas et al., 2011; Wiggs et al., 2012). A remaining challenge is to determine and prove the specific roles of the glaucoma-associated genes in disease pathogenesis.

Use of mouse models is a viable approach for dissecting the complex genetics of IOP elevation and glaucoma for several reasons. First, there are striking similarities between the human and mouse ocular drainage structures. The development and organization of these structures, including the trabecular meshwork, closely resemble each other in mice and humans (Civan and Macknight, 2004; Gould et al., 2004; Smith et al., 2001; Tamm, 2013). Second, the dynamics of aqueous humor production and outflow are similar between mice and humans (Aihara et al., 2003; Crowston et al., 2004). Thus, mice provide a valuable model for validating genes associated with a risk of high IOP and glaucoma in human studies. Furthermore, mice provide an important experimental platform that can be used to elucidate molecular mechanisms and discover new glaucoma genes.

Glaucoma gene discovery using mice requires phenotypically affected mouse strains that are often inbred (Anderson et al., 2002; Chang et al., 1999; John et al., 1998) or chemically mutagenized (Cross et al., 2014; Gould et al., 2007; Lachke et al., 2011; Nair et al., 2011), or mice with other induced or spontaneous mutations (Buys et al., 2014; Chang et al., 2001; Mao et al., 2011). This strategy of using mutant mice with glaucoma-relevant phenotypes has been effective for identifying causative genes and understanding the underlying disease mechanism. For example, the inbred mouse strain DBA/2J (D2) is a widely used glaucoma model. These mice spontaneously develop a chronic age-related glaucoma. Mutations in two D2 genes [tyrosinase-related protein 1 (*Tyrp1*) and glycoprotein (transmembrane) nmb (*Gpnmb*)] have been shown to induce a depigmenting iris disease (hereafter referred to as iris disease) characterized by abnormal liberation of iris pigment into the ocular anterior chamber (Anderson et al., 2002; Chang et al., 1999). After pigment dispersion, these mice develop high IOP and pressure-induced glaucomatous neurodegeneration (Libby et al., 2005a). The D2 model has contributed considerably to our understanding of mechanisms that underlie pigment dispersion, IOP elevation and optic nerve degeneration (Howell et al., 2011, 2007a,b, 2012; Libby et al., 2005b; Nickells et al., 2012).

A variety of animal models with different genetic causes and genetic backgrounds are needed to fully understand the complex etiology of glaucoma. Given their relative affordability and experimental power, a variety of new mouse models are needed for both gene identification and validation, as well as for mechanistic studies. A first step to gene discovery using mice is to characterize disease phenotypes. Here, we characterize an inbred mouse strain, YBR/EiJ (YBR), as a new model of glaucoma. Identifying genetic factors contributing to glaucoma in YBR mice has potential to further our understanding of mechanisms that underlie high IOP and glaucoma.

## RESULTS

### YBR mice exhibit iris stromal atrophy and pigment dispersion

**Fig. 1.**
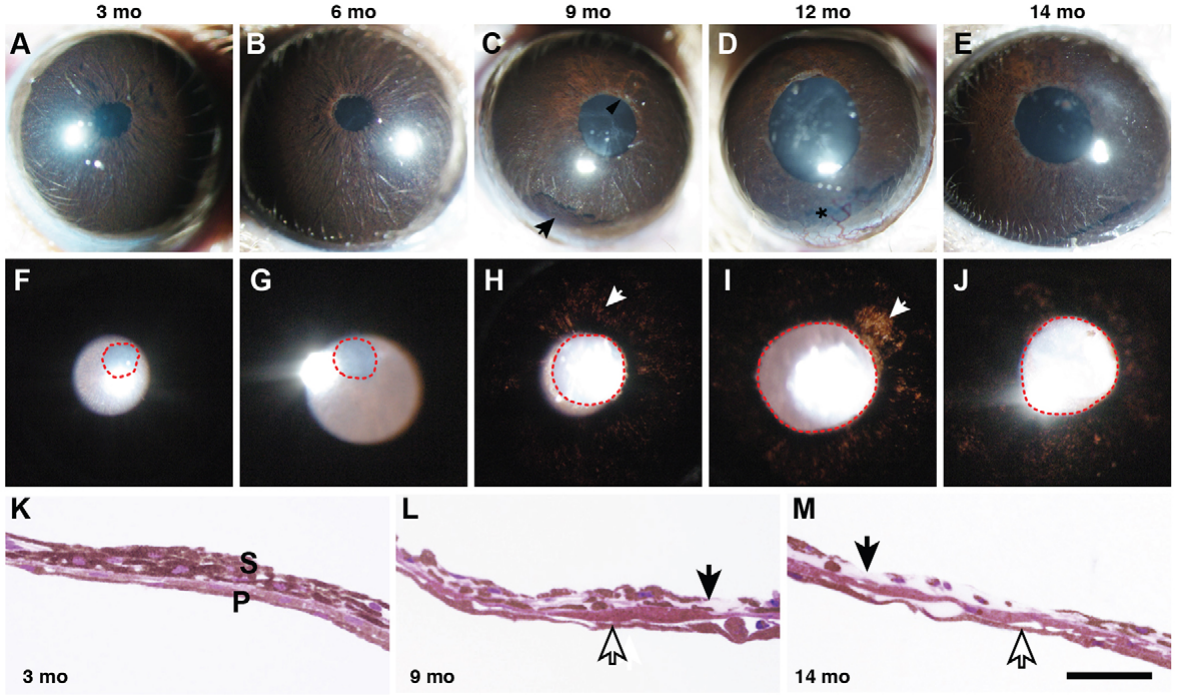
**YBR mice exhibit a form of pigment-dispersing iris disease.** YBR mice exhibit a progressive iris disease with age, characterized by iris stromal atrophy and pigment dispersion. (A-E) The top row of panels shows broad-beam illumination of YBR eyes at each of the indicated ages to assess the presence of dispersed pigment within the anterior chamber and iris stromal morphology. Mo, months. (F-J) The bottom row of panels shows transillumination to assess the degree of iris depigmentation, as revealed by areas where reflected light passes through the iris (white arrows). Pupils are outlined by red dotted lines. With transillumination, reflected light passes through the iris, after reflecting off the posterior pole of the eye. Normally, this reflected light would be blocked by the iris. (A,F) Three-month-old YBR eyes have normal iris morphology with clearly evident iris details. (B,G) By 6 months, mutant mice exhibited mild iris disease characterized by the mild accumulation of iris pigment in the peripupillary region. (C,H) By 9 months, YBR eyes exhibited iris stromal atrophy characterized by thinning of the iris stroma and peripupillary atrophy (black arrow head, C) and mild transillumination defects (white arrow, H). The degree of iris atrophy progressively worsened with age. Accumulation of dispersed pigment was greatest in the inferior angle (black arrow, C) owing to gravity. (D,I) At 12 months, some of the YBR eyes exhibited vascularization of the cornea (black asterisk, D), and most eyes showed worsening transillumination defects (white arrow, I). (K) In a young YBR mouse, the iris was well developed and had an intact stroma (S) and pigment epithelium (P). (L) The iris of an old YBR mouse exhibited moderately atrophied stroma (black arrow) and intact pigment epithelium (open arrow). (M) In severe cases, the stroma was largely atrophied with areas where it was essentially non-existent (black arrow). The iris pigment epithelium of these mice remained remarkably intact considering the overall condition of the iris (open arrows). Scale bar: 50 µm.

YBR mice were aged and, using a slit lamp, their eyes were assessed for the iris phenotype and pupillary abnormalities every 2 to 3 months between 3 and 16 months of age. YBR mice develop an age-related iris disease that primarily affects the iris stroma and resembles the iris stromal atrophy phenotype of DBA/2J mice (Chang et al., 1999) (Fig. 1A-J). By 6 months of age, a mild iris stromal atrophy was evident (Fig. 1B). With increasing age, the iris depigmentation became more prominent and transillumination defects were evident (areas of depigmented iris that allow light to pass through, Fig. 1). Iris depigmentation was further corroborated by a histological examination of the iris (Fig. 1K-M). The iris depigmentation and cell loss was restricted to the iris stroma with no obvious loss of iris pigment epithelium cells (Fig. 1L,M).

### YBR mice develop elevated IOP

**Fig. 2.**
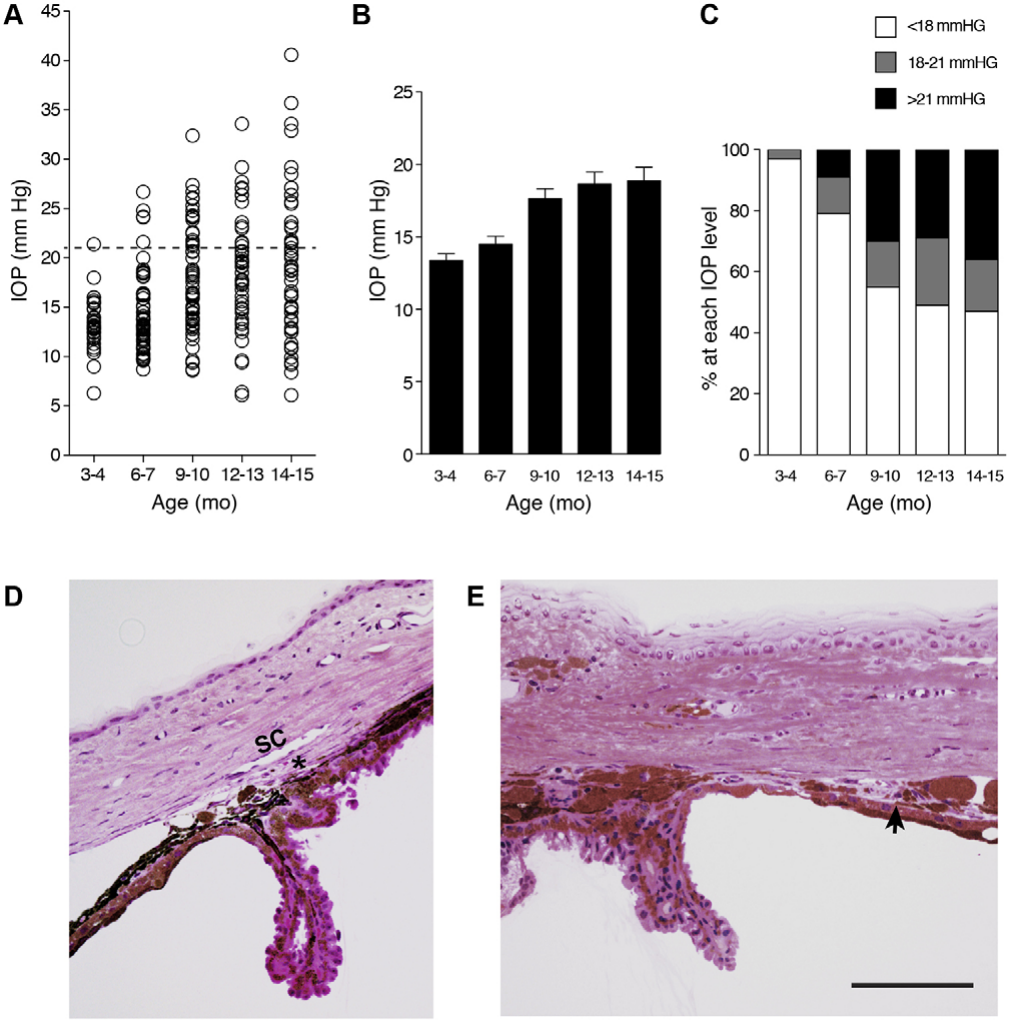
**YBR mice exhibit elevated IOP subsequent to iris disease.** (A) The IOP values at each of the indicated ages are represented as a scatter plot. Dashed line indicates IOP at 21 mmHg (regarded as glaucoma relevant). Mo, months. (B). The same IOP values are also represented as a bar graph of the mean IOP±s.e.m. (C) Percentage of eyes with IOP in each indicated pressure range at the indicated ages. All groups contained at least 30 mice, and included both males and females in similar proportions. With age, YBR eyes developed high IOP. By 12-13 months, the IOP values leveled out as there was no further increase in mean IOP with advancing age. (D) Young YBR mice have normal open iridocorneal angles. The angle has a well-formed trabecular meshwork (asterisk) and an open Schlemm's Canal (SC). (E) In an aged 14-month-old YBR mouse, there was variability in the angle morphology. Some angle regions exhibited anterior synechia (arrow) occluding the drainage tissue. Schlemm's canal and the trabecular meshwork were occluded and not readily identifiable. Scale bar: 100 µm.

We next determined the IOP of YBR mice between 3 and 15 months of age. Because 95% of values fell within 2 standard deviations (s.d.) of the mean and 18 mmHg is 2 (s.d.) from the mean of young YBR mice, we regard an IOP value>18 mmHg as high. As is common, we regard IOP values greater than 21 mmHg as glaucoma relevant, owing to the further increased risk of developing glaucoma at these values. The number of YBR mice developing high IOP increased starting at 6 to 7 months. With advancing age there was a further increase in the proportion of mice with high IOP (Fig. 2A-C). The iris atrophy and pigment dispersion preceded the IOP elevation and are likely to contribute to inefficient aqueous humor drainage. Histological analyses showed that following deterioration of the YBR iris stroma, the released pigment and cellular debris initially accumulated at the iridocorneal angle. The angle contains the ocular drainage structures (trabecular meshwork and Schlemm's canal) and runs 360° around the limbus of the eye. With age, the pigment accumulation induced damage to the angle, which progressed to the formation of focal tissue adhesions (anterior synechiae and angle closure) that block aqueous humor drainage (Fig. 2D,E).

### Glaucomatous neurodegeneration in YBR eyes

**Fig. 3.**
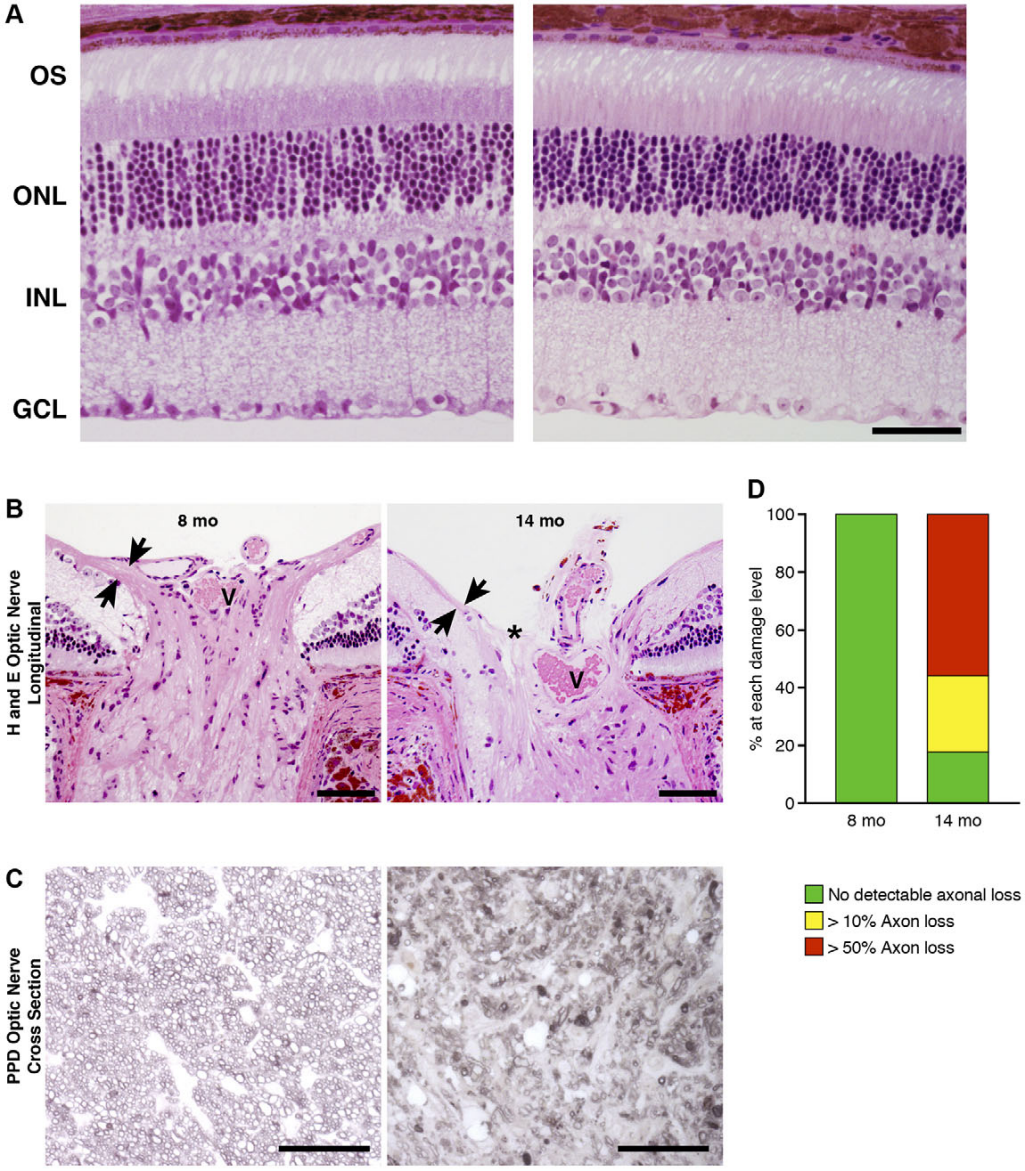
**YBR mice develop glaucomatous neuropathy.** (A) Retinas of representative YBR mice. Eight-month-old (without any sign of glaucoma, left panel) and 14-month-old (exhibiting glaucoma, right panel) retinas are shown. Both retinas have a normal outer segment (OS), outer nuclear layer (ONL) and inner nuclear layer (INL). However, the retina from the14-month-old YBR mouse has fewer retinal ganglion cells in the ganglion cell layer (GCL). (B) YBR eyes at 8 months have a normal optic nerve head characterized by a thick nerve fiber layer entering the optic nerve (arrows facing each other) and a central vessel (V). By 14 months, YBR eyes develop advanced optic nerve excavation (asterisk) and extreme thinning of the nerve fiber layer (arrows facing each other). Scale bars: 50 µm. (C) Optic nerve degeneration was analyzed using PPD-stained optic nerve cross sections. Nerves from 8-month-old mice (representative image, left panel) had no detectable axonal damage, and axons had a clear axoplasm and darkly stained myelin sheaths. By 14 months, however, nerves had severe damage and showed extensive axon loss and glial scarring (representative image, right panel). Scale bars: 50 µm. (D) A bar graph representing the degree of optic nerve damage at the indicated ages. There were at least 30 eyes in each group with both sexes in similar proportions. At 8 months, none of the YBR eyes exhibited glaucomatous neurodegeneration. By 14 months, about 60% of YBR eyes exhibited severe glaucomatous nerve damage and axonal loss.

To determine whether high IOP leads to optic neuropathy, we performed a histological assessment of the retinas and optic nerves of YBR eyes. Characteristic of glaucoma, the retinal layers of the older YBR eyes were indistinguishable from those of the young YBR eyes, except for the loss of retinal ganglion cells (RGCs) and thinning of the nerve fiber layer, which contains RGC axons (Fig. 3A). Aged YBR eyes also displayed optic nerve excavation and remodeling, a classic hallmark of glaucomatous neurodegeneration (Fig. 3B). Assessment of RGC axons in optic nerve cross sections at 8 months (an age before significant elevation in IOP was detected) demonstrated that essentially all axons had a healthy morphology (Fig. 3D). By 14 months, about 60% of YBR eyes had severe axon loss with extensive gliosis and optic nerve damage (Fig. 3C,D). Taken together, our data suggest that YBR mice develop a pressure-induced optic nerve degeneration that is characteristic of glaucoma.

### Identification of genetic loci contributing to pigment dispersion and IOP elevation

To identify genetic factors that contribute to the pigment-dispersing iris disease and high IOP, we performed gene-mapping experiments. For these experiments, the glaucoma-susceptible YBR strain of mice was crossed to C57BL/6J (B6) mice, a control strain that does not develop glaucoma. The F1 progeny were backcrossed to YBR to allow random segregation of chromosomal regions in the resulting N2 progeny. To determine if iris disease was present and correlated with elevated IOP, N2 progeny underwent examination with a slit lamp and IOP measurements between the ages of 8 and 17 months. N2 progeny were genotyped using single nucleotide polymorphic (SNP) marker analysis.

**Fig. 4.**
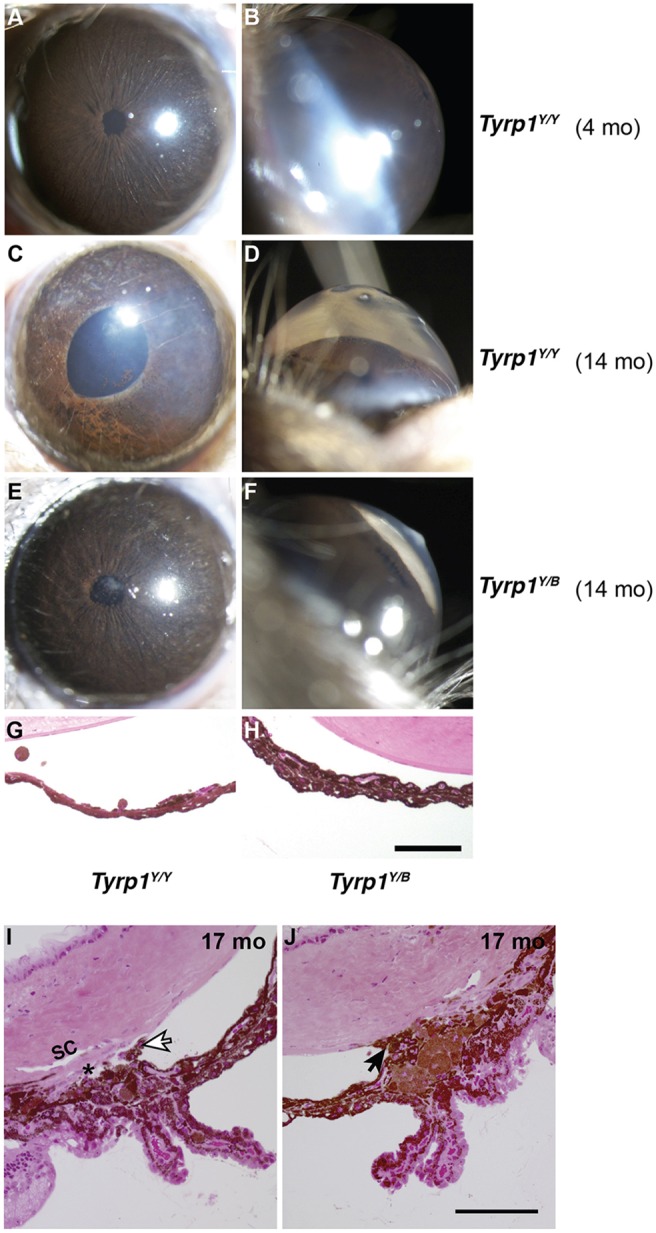
**Iris disease and high IOP are genetically distinct.** (A-F) Representative images (broad-beam illumination) of young control YBR mice (4 months) and old affected N2 mice (14 months) that are heterozygous or homozygous for the YBR-derived *Tyrp1^b^* allele. Y and B denote YBR and B6 alleles, respectively. (A-D) Mice that are homozygous for *Tyrp1^b^* (*Tyrp1^Y/Y^*) developed age-related iris stromal atrophy and their anterior chambers became deepened owing to high IOP. (E,F) Some heterozygous mice (*Tyrp1^Y/B^*), despite having an intact iris, developed high IOP (range 21.1-32.1 mmHg) and exhibited deep anterior chambers. This suggests that iris disease and high IOP are genetically separable. (G,H) Representative image of the iris from N2 mice (14 months) that are homozygous for the chromosome 17 (Y/Y) locus but are either homozygous (*Tyrp1^Y/Y^*) or heterozygous (*Tyrp1^Y/B^*) for *Tyrp1.* (I) Representative images from a histological evaluation of angles spanning much of the thickness of the eyes of *Tyrp1^Y/B^* mice (Materials and Methods). Despite high IOP, a normal iris and open angle was observed in most regions of this eye. White arrow indicates an iris process, a structure known to be in close proximity to the drainage tissue even in normal mouse eyes. Despite the sectioning-based distortion that displaced the iris processes towards the drainage structures, this section clearly shows a normal mouse open angle. (J) Although >60% of the extent of the angle was open in each of these eyes, there were local regions in which the angle was closed owing to anterior synechia (arrow). Local angle closure is common in non-glaucomatous mouse strains with normal IOP at this age, which is likely to be due to their naturally very narrow angles. Scale bars: 100 µm.

Slit-lamp examination of the N2 progeny revealed a spectrum of severity of iris phenotypes that broadly formed two groups: mice with normal irises, and mice with prominent iris disease characterized by stromal atrophy and abnormal pigment dispersion (Fig. 4A-D). Only mice with a brown coat exhibited the iris disease phenotype, but only 86% of mice with a brown coat had the disease. A recessive *Tyrp1^b^* allele on chromosome 4 is responsible for the brown coat color present in various inbred mouse strains (Bennett et al., 1990), and we have previously demonstrated this *Tyrp1^b^* allele as responsible for the iris stromal atrophy phenotype in D2 mice (Anderson et al., 2002; Chang et al., 1999). Our allele-specific and sequence analyses confirmed that the YBR strain had the *Tyrp1^b^* allele. The *Tyrp1^b^* allele encodes a mutant protein containing two missense mutations resulting in the Cys to Tyr substitution at position 86 and the Arg to His substitution at position 302, compared with the *Tyrp1* allele of C57BL/6J mice. *Tyrp1^b^* results in a brown coat. It has been hypothesized that *Tyrp1^b^* alters the melanosomal membrane, allowing cytotoxic intermediates of pigment production to induce the iris stromal atrophy disease (Anderson et al., 2002). These findings suggest that *Tyrp1^b^* underlies the iris disease in YBR eyes.

**Fig. 5.**
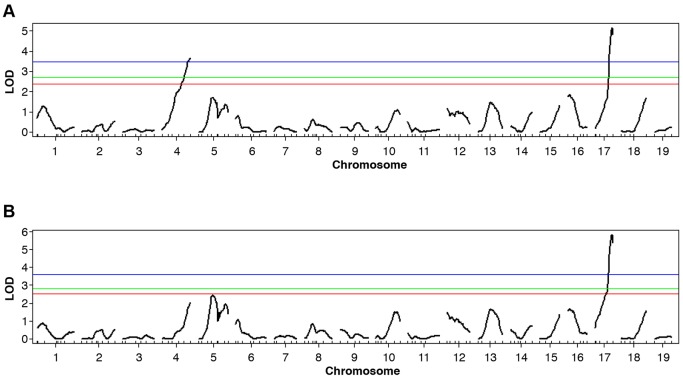
**QTL analyses identify genetic regions contributing to high IOP in YBR mice.** (A) A genome-wide scan using IOP values as a quantitative trait in N2 mapping progeny identified two significant QTLs: on chromosome 4 and chromosome 17. The blue, green and red lines indicate the significance thresholds at 1%, 5% and 10%, respectively. Both the chromosome 4 and chromosome 17 regions show LOD scores above the 1% threshold. (B) A genome-wide scan of only black mice (*Tyrp1^Y/B^*, with no iris disease) identified a significant QTL on chromosome 17.

Consistent with IOP elevation caused by the depigmenting iris disease and angle closure, high IOP was common in eyes with iris disease. Interestingly, however, some mice without any sign of iris disease developed high IOP (Fig. 4E,F). It is well established that within an eye, the angle can have local regions that are either normal or abnormal (Chang et al., 2001; Libby et al., 2003; Smith et al., 2000). Unlike the mice with extensive iris disease and secondary angle closure, a subset of progeny developed a high IOP that was independent of the iris disease (normal iris, Fig. 4H) but coincided with largely open angles as assessed by histology (>60% open or normal, Fig. 4I,J). Thus, iris disease and high IOP are sometimes genetically separable, indicating that factor(s) other than iris disease might contribute to high IOP. We, therefore, performed quantitative trait locus (QTL) analysis to identify genetic loci contributing to high IOP. Our QTL analysis using IOP values as a continuous trait detected two important chromosomal regions: one on chromosome 4 (likely to be *Tyrp1^b^*) and one on chromosome 17 (Fig. 5A, Table 1). Next, and to delineate the effect of iris disease from other factors contributing to high IOP, we performed a QTL analysis on a cohort of mice with a black coat; hence, these mice are only heterozygous for the *Tyrp1^b^* region and do not exhibit iris disease. This strategy increases the likelihood of detecting additional genes acting independently of the iris disease that could have been missed in our earlier analysis. This QTL analysis revealed that only the YBR chromosome 17 locus significantly contributes to the generation of high IOP (Fig. 5B). This statistically demonstrates that a gene within the chromosome 17 locus contributes to high IOP in the absence of the iris disease.

**Table 1.**
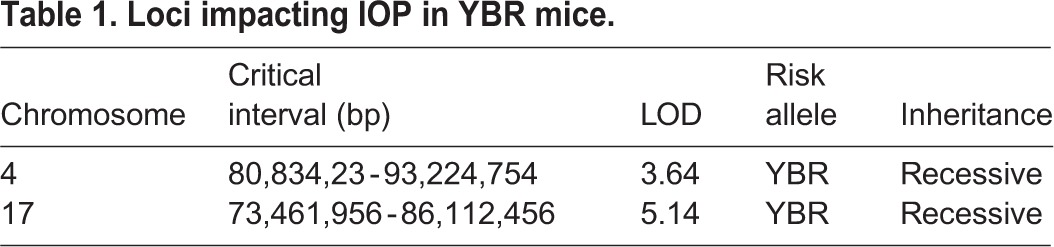
**Loci impacting IOP in YBR mice.**

### Iris disease and the YBR chromosome 17 locus induce high IOP

**Fig. 6.**
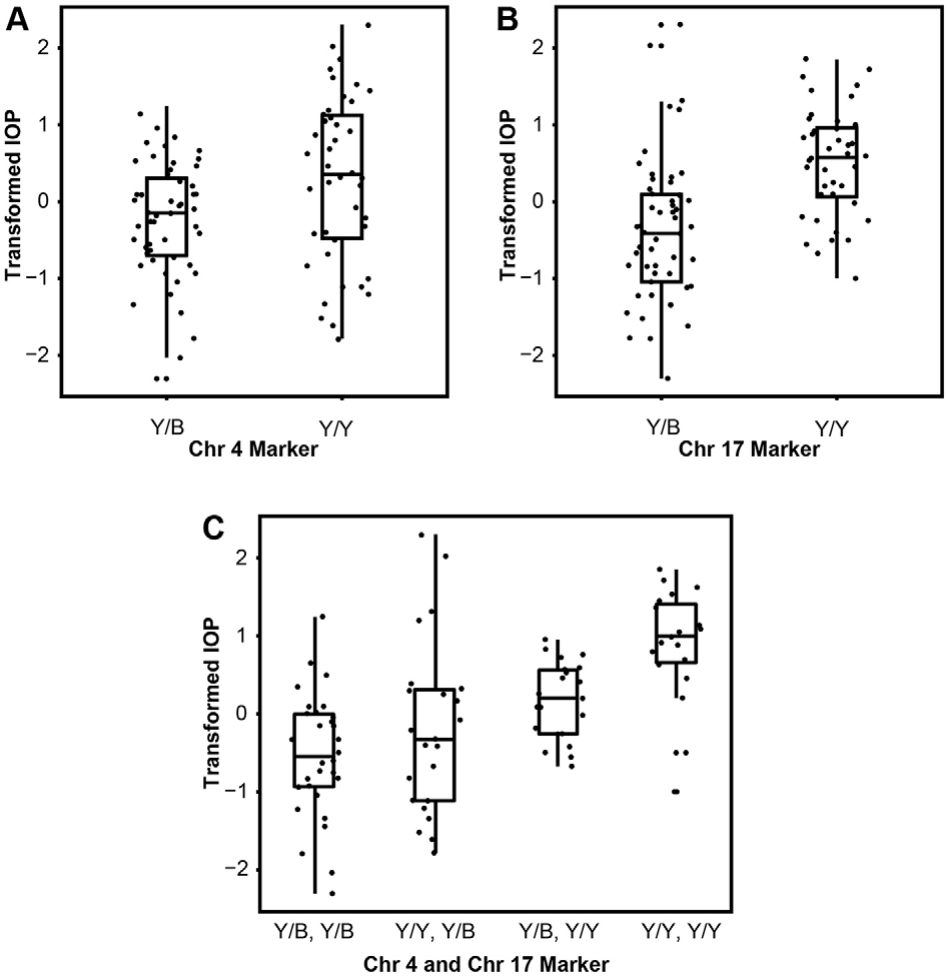
**Factors independent of iris disease contribute to high IOP.** Graphs showing the relationship between IOP (transformed values, see Materials and Methods) and the genotypes at the two significant QTL markers (at chromosome 4 and chromosome 17). (A,B) Mice homozygous for either a recessive YBR chromosome 4 or YBR chromosome 17 locus have significantly higher IOP than mice carrying their respective heterozygous alleles (*P*-value=4.239×10^−3^ for YBR chromosome 4 comparison and *P*-value=2.125×10^−6^ for YBR chromosome 17 comparison). (C) Mice homozygous for both the recessive YBR chromosome 4 and YBR chromosome 17 loci, respectively (Y/Y, Y/Y), have significantly higher IOP compared to mice carrying heterozygous alleles on both loci (chromosome 4 Y/B, chromosome 17 Y/B) or a recessive allele on any one of the loci (chromosome 4 Y/Y, chromosome 17 Y/B or chromosome 4 Y/B, chromosome 17 Y/Y). *P*=1.05×10^−7^ for ‘chromosome 4 Y/Y, chromosome 17 Y/Y’ vs ‘chromosome 4 Y/B, chromosome 17 Y/B’ comparison. *P*=5.75×10^−4^ for ‘chromosome 4 Y/Y, chromosome 17 Y/Y’ vs ‘chromosome 4 Y/Y, chromosome 17 Y/B’ comparison and *P*=1.07×10^−3^ for ‘chromosome 4 Y/Y, chromosome 17 Y/Y’ vs ‘chromosome 4 Y/B, chromosome 17 Y/Y’ comparison. Y and B denote YBR and B6 alleles, respectively. Chr, chromosome. Graphs are boxplots, where boxes represent data within the first and third quartiles, the whiskers represent the median values, the ends of vertical lines represent the minimum and maximum values, and the dots represent the actual data points staggered horizontally to avoid any overlaps.

Further analysis of the implicated chromosomal regions (YBR chromosome 4 and chromosome 17) using correlation analysis (linear regression) demonstrated that the mice homozygous for recessive YBR loci (Y/Y) on either chromosome 4 or chromosome 17 develop significantly higher IOP compared to mice heterozygous for each of the YBR loci (Y/B) (Fig. 6A,B). To further understand the role of the YBR chromosome 4 and chromosome 17 loci, we tested whether these two loci interact to induce high IOP using an ANOVA full model. Results of our analysis demonstrated that the peak markers on chromosome 4 and chromosome 17 do not interact (*P*=0.285). Next, we employed an additive model to test the combined effect of markers on chromosome 4 and chromosome 17 on IOP outcome. Exclusion of either the chromosome 4 or chromosome 17 region from the additive model caused a significant decrease in the magnitude of IOP and frequency of mice developing high IOP (*P*=0.0042 for the effect of chromosome 4 exclusion and *P*=2.2125×10^−5^ for the effect of chromosome 17 exclusion). In addition, a correlation analysis demonstrated that mice carrying both the recessive YBR chromosome 4 and chromosome 17 loci develop significantly higher IOP compared to mice heterozygous for a recessive allele in any one of the loci (Fig. 6C), supporting that each gene has recessive effects.

## DISCUSSION

Glaucoma is a complex and heterogeneous disease affected by many genetic factors. Thus, the study of a variety of genetically diverse glaucoma models is warranted. Here, we introduce and characterize the YBR/EiJ mouse strain as a new model of glaucoma. YBR mice develop an age-related pigment-dispersing iris disease. They subsequently develop high IOP and hallmarks of glaucomatous neurodegeneration. Additionally, we identify genetic loci that contribute to iris disease and high IOP in the YBR strain. These mice will be important for identifying new genes and mechanisms that cause high IOP and glaucoma.

### Iris stromal atrophy in YBR mice has similarities to essential iris stromal atrophy

The iris disease in YBR mice is characterized by iris stromal atrophy (Fig. 1). Some aspects of the YBR phenotype have strong similarities to human essential iris atrophy (EIA). During EIA, iris stromal atrophy is often accompanied by formation of abnormal adherence of the iris to cornea and trabecular meshwork (peripheral anterior synechiae; PAS), and by abnormal proliferation of corneal endothelium over the trabecular meshwork and iris (iridodocorneal endothelial syndrome; ICE) (Shields, 1979, 1997; Shields and Bourgeois, 1996). This leads to blockage of aqueous humor access to the drainage pathway (secondary angle closure) and elevation of IOP. In YBR eyes, the released iris stromal pigment and cellular debris accumulate within the ocular drainage structures, subsequently leading to angle closure, IOP elevation and glaucoma. The etiology of EIA is not well understood. It is suggested to be primarily an acquired disease, mediated by viral infection causing subclinical inflammation around the corneal endothelium. However, some familial cases of EIA have been reported, and an interplay of genetic and environmental factors is likely to participate in disease pathogenesis (Shields, 1979)**.**

### Genetics of iris stromal atrophy

Phenotypic analyses of the segregating progeny from our mapping cross demonstrate that the iris disease in these mice is linked to a gene responsible for the brown coat color. The *Tyrp1^b^* allele underlies a brown coat color in various inbred mouse strains (Bennett et al., 1990). Both YBR and D2 mice share the same allele of *Tyrp1* (*Tyrp1^b^*). Therefore, as is well established for glaucomatous D2 mice (Anderson et al., 2002; Chang et al., 1999), the *Tyrp1^b^* allele is likely to be responsible for iris stromal atrophy and IOP elevation in YBR mice. However, compared to the D2 mice, the degree of iris depigmentation was less severe in age-matched YBR mice. This is likely to be because, in D2 mice, a digenic combination of mutant *Tyrp1* and mutant *Gpnmb* is responsible for earlier onset and a more severe iris disease, which impacts both the iris pigment epithelium and the iris stroma (Chang et al., 1999). However, in YBR mice, the iris pigment epithelium remained largely intact, and only the iris stroma degenerates (Fig. 1K-M). Our genetic analysis failed to detect any additional factor(s) that contribute to iris disease in the YBR mice. Moreover, YBR mice have a wild-type form of *Gpnmb*, ruling it out as a factor that contributes to progression of their iris disease (our unpublished data). It remains possible that, in YBR mice, *Tyrp1^b^* interacts with multiple genes that have small effects on the disease phenotype but that were undetected in our analysis. Interestingly, only 86% of homozygous *Tyrp1^b^* N2 mice in the current study developed iris disease, compared to the fully penetrant effect of *Tyrp1^b^* on iris disease observed in all of our previous studies on different genetic backgrounds (Anderson et al., 2001; Chang et al., 1999; John et al., 1998), suggesting that other modifier gene(s) could have an influence.

### Genetics of high IOP and glaucoma

Our results indicate that iris disease plays an integral role in the pathogenesis of IOP elevation in YBR mice (Fig. 6A). However, a distinct YBR locus on chromosome 17 induces high IOP independent of the iris disease phenotype (Fig. 6B). Based on statistical modeling, we interpret our results as follows: the YBR chromosome 4 locus primarily dictates the iris disease phenotype, and the deposited pigment and debris impact the drainage tissue, contributing to angle closure and high IOP. The YBR chromosome 17 locus acts through a distinct mechanism (not involving iris disease) to induce high IOP. Although these loci underlie distinct pathogenic processes, they act in an additive fashion to promote an elevation of IOP.

Interestingly, some of the mice used to map the genes exhibited no iris disease (homozygous for the YBR chromosome 17 locus but not for the YBR chromosome 4 locus) had angles that were open to a large extent (>60% of analyzed angle open and <40% closed or damaged, Fig. 4). Local angle closure and pigment accumulation are common in old mice that have normal IOP (our personal observation). Additionally, in a previous mouse study, only eyes with severe angle abnormalities, extending around 80% or more of the angle, had high IOP (Chang et al., 2001). In agreement with the mouse data, in human eyes with angles damaged by trauma, eyes with similarly extensive angle damage frequently have high IOP but eyes with less extensive damage (<240° or <67% of any angle damaged) do not (Alper, 1963; Kaufman and Tolpin, 1974). Based on these observations, it is possible that a gene within the chromosome 17 locus contributes to elevation of IOP by directly impacting the drainage structures rather than through an indirect effect (pigment accumulation or angle closure). Therefore, the YBR chromosome 17 gene might have relevance for the pathogenesis of open-angle glaucomas.

In summary, we introduce a new and needed inherited model of glaucoma, and have identified a new genetic locus that induces high IOP even in eyes with no iris disease and largely open angles. Current efforts are focused on identifying the causal gene and mutation.

## MATERIALS AND METHODS

### Animal husbandry

Mice were obtained from the Jackson Laboratory. All experiments were conducted in accordance with the Association for Research in Vision and Ophthalmology's statement on the use of animals in ophthalmic research. The Jackson Laboratory's Institutional Animal Care and Use Committee approved all procedures described here. The YBR/EiJ mice were maintained on a NIH31 diet (6% fat) and HCl acidified water (pH 2.8-3.2). To avoid obesity, C57BL/6J (B6) mice and progeny used for gene mapping were fed with the same NIH31 diet but with a 4% fat content. Diets were provided *ad libitum*. Mice were housed in cages with pine wood shavings as bedding and covered with polyester filters. The cages were maintained in an environment kept at 21°C with a 14-h light and 10-h dark cycle.

### Clinical examination

Eyes were examined with a slit-lamp biomicroscope and photographed with a 40× objective lens. All photographs were taken using identical camera settings. Phenotypic evaluation included iris structure, pupillary abnormalities, cataracts and the overall dimensions of the anterior chamber. Iris disease was evaluated by indices of iris atrophy and dispersed pigment following previously described criteria (John et al., 1998). Transillumination assays were performed to assess the extent of iris depigmentation. Briefly, a narrow beam of light passed through the pupil is reflected back through depigmented areas of iris tissue and are visualized as reddish light. Examination of at least 30 mice of each genotype at 3, 6, 9, 12 and 14 months of age was performed. All cohorts included male and female mice. Additionally, smaller groups of mice (10-15) were analyzed every month between 3 and 16 months of age.

### IOP Measurement

IOP was measured using the microneedle method, as previously described in detail (John et al., 1998). Briefly, mice were anesthetized with an intraperitoneal injection of a mixture of ketamine (99 mg/kg of bodyweight; Ketalar, Parke-Davis, Paramus, NJ) and xylazine (9 mg/kg of bodyweight; Rompun, Phoenix Pharmaceutical, St. Joseph, MO) before IOP assessment – a procedure that does not alter IOP in the experimental window (Savinova et al., 2001). All cohorts included male and female mice. In all our experiments, B6 mice were used along with experimental mice as a methodological control to ensure proper equipment calibration and function.

### Optic nerve assessment

The optic nerves were collected from mice within 48 h of IOP measurement. Each age group contained samples from males and females, as well as left and right nerves. Intracranial portions of the optic nerves were dissected and assessed for glaucomatous damage as previously described (Howell et al., 2007a). Briefly, the optic nerve cross sections were stained with para-phenylenediamine (PPD) and examined for glaucomatous damage. PPD stains the myelin sheaths of a healthy axon, but also stains both the myelin sheaths and the axoplasm of sick or dying axons, thus allowing very sensitive detection and quantification of axon damage and loss, according to previously validated and published criteria (Howell et al., 2007a, Howell et al., 2012).

### Assessment of the drainage tissue

Hematoxylin and eosin (H&E)-stained ocular sections from three mapping progeny (six eyes) with a black coat (heterozygous for the *Tyrp1^b^* allele) and exhibiting high IOP were assessed to determine if the angles were open or closed as a result of synechiae. We evaluated ten similarly spaced (15 to 20 μm) sections for three ocular regions (peripheral, mid-peripheral and central). Both angles of a section were considered for evaluation. Thus, a total of 60 angle locations were analyzed in each eye (ten sections×two angles×three regions). Thereafter, we calculated the percentage of angles that were open.

### Gene mapping and QTL studies

To map genes controlling iris disease and high IOP, glaucoma-susceptible YBR mice were crossed to glaucoma-resistant B6 mice. All the F1 progeny exhibited no signs of iris disease or high IOP. We utilized a backcross strategy by crossing F1 generation progeny to the parent YBR strain. A total of more than 90 N2 progeny were aged up to 17 months, and clinical examination of their eyes was performed every 2 months between 3 and 17 months. IOP was measured at multiple time points between the ages of 8 and 17 months. For each test animal, the highest IOP value detected at any age was considered for subsequent QTL analysis. Initial genotyping was performed using over 116 genome-wide single nucleotide polymorphic markers that differentiate the YBR from the B6 genome and spaced approximately at about 20-Mb intervals (SNPs, KBioscience, UK). Y and B denote YBR and B6 alleles, respectively, throughout the manuscript. We performed a genome-wide one-dimensional QTL scan to identify the chromosomal loci regulating IOP. R/QTL software version 1.14-2 was used for QTL analysis (Broman et al., 2003). Inverse transformed IOP values were used in the analysis because they showed a normal distribution. Pseudo-markers were generated at 2-cM spacing for each chromosome, and a whole genome scan was performed using 256 imputations. One thousand permutations were performed to determine the thresholds for QTL detection. Three thresholds at 1%, 5% and 10% were calculated from the permutation results. A QTL with LOD score above the 1% threshold was classified as a strong QTL, whereas those at or above 10% were classified as a suggestive QTL. Genotyping quality assessment was performed by evaluating the recombination fraction plot and map plot; fine mapping of a crucial YBR chromosome 17 region (69 Mb to 86 Mb) was performed using MIT markers distinguishing YBR from B6 genome (NCBI Build 37).

### Correlation analysis

We used rank-Z-transformed IOPs for the following statistical analysis. We tested for the interaction effect between the markers on chromosome 4 and chromosome 17 on IOP. For this purpose, we used a full model (IOP∼chromosome4+chromosome17+chromosome4:chromosome17), including additive main effects and their interactions, and a reduced model (IOP∼chromosome4+chromosome17) with only additive main effects. We compared these two models using ANOVA and evaluated the significance of interaction with an F-test using R software. After finding no interaction effect, we used the additive model (IOP∼chromosome4+chromosome17) and tested for each main effect by dropping the corresponding term and computed the F-statistic. In addition, we performed *t*-tests (two tailed) to compare mice with various genotypic combinations of the implicated YBR chromosome 4 and chromosome 17 loci. For data in Fig. 6C, a significant *P*-value threshold of 0.05 is equivalent to 0.05/6=0.0083 by using Bonferroni's correction for multiple comparisons (we performed six pairwise comparisons).
